# TRIM14 Regulation of Copper Homeostasis and Cuproptosis: A New Strategy to Overcome Chemoresistance in Glioblastoma

**DOI:** 10.3390/biomedicines13123085

**Published:** 2025-12-15

**Authors:** Jianyong Wang, Enhao Zhang, Siqi Chen, Haifeng Wang, Yi Huang, Wenting Lan

**Affiliations:** 1 Ningbo Key Laboratory of Nervous System and Brain Function, Department of Neurosurgery, The First Affiliated Hospital of Ningbo University, Ningbo 315010, China; fyywangjianyong@nbu.edu.cn (J.W.); 2311140229@nbu.edu.cn (E.Z.); 2311140031@nbu.edu.cn (S.C.); fyywanghaifeng10630@nbu.edu.cn (H.W.); 2 Ningbo Key Laboratory of Nervous System and Brain Function, Department of Radiology, The First Affiliated Hospital of Ningbo University, Ningbo 315010, China

**Keywords:** glioblastoma, cuproptosis, temozolomide, TRIM14, ATP7A, chemosensitivity

## Abstract

**Background**: Glioblastoma (GBM) is an aggressive primary brain tumor characterized by limited therapeutic options and poor prognosis. Temozolomide (TMZ) remains the standard chemotherapy; however, its effectiveness is often hindered by the development of acquired resistance. Cuproptosis, a recently identified copper-dependent form of regulated cell death, has emerged as a potential therapeutic target. The synergistic effects of TMZ and copper, as well as the molecular mechanisms underlying their combined action, remain unclear. This study aimed to investigate the role of tripartite motif-containing protein 14 (TRIM14) and its downstream effector ATP7A in mediating TMZ- and copper-induced cuproptosis in glioma. **Methods**: We employed in vitro cellular assays, in vivo xenograft models, bioinformatic analysis, immunofluorescence staining, Western blotting, and co-immunoprecipitation experiments to examine the functional involvement of TRIM14 and ATP7A during combined TMZ and copper chloride (CuCl_2_) treatment. Intracellular copper levels and cuproptosis markers, including Dihydrolipoamide S-acetyltransferase (DLAT), were assessed to evaluate copper-dependent cytotoxicity. **Results**: TMZ combined with CuCl_2_ markedly enhanced cuproptosis in glioma cells, as evidenced by increased DLAT expression and elevated intracellular copper accumulation. This combination treatment significantly suppressed TRIM14 expression, downregulated the TRIM14–ATP7A axis, and inhibited non-canonical NF-κB signaling. Co-immunoprecipitation assays further revealed a potential interaction between TRIM14 and ATP7A, suggesting that TRIM14 may modulate ATP7A stability or activity. **Conclusions**: Our findings indicate that TMZ and copper synergistically induce cuproptosis in GBM by disrupting the TRIM14–ATP7A regulatory axis and promoting intracellular copper accumulation. Targeting TRIM14 or ATP7A to enhance cuproptosis may represent a promising therapeutic strategy to overcome TMZ resistance and improve clinical outcomes in GBM patients.

## 1. Introduction

Glioma, the predominant malignant brain tumor, presents significant treatment difficulties because of its aggressive spread and inherent therapy resistance [1]. The median survival for patients with GBM receiving standard treatment—including surgical resection, chemotherapy, and radiotherapy—is only 14.6 months [2]. According to the World Health Organization (WHO) classification of central nervous system tumors, gliomas are graded on a scale from I to IV based on malignancy. Grades I–II are defined as low-grade gliomas (LGG), while grades III–IV are classified as high-grade gliomas (HGG). In this categorization scheme, glioblastoma (GBM, WHO stage IV) is deemed the most aggressive form of glioma, characterized by its extreme invasiveness and significant heterogeneity [3]. Currently, a variety of treatment modalities—including surgical resection, adjuvant radiotherapy, chemotherapy, immunotherapy, photodynamic therapy, and tumor-treating fields—have been widely applied in glioma management; however, patient prognosis remains unsatisfactory [4].

Copper-induced cell death, or cuproptosis, represents a recently discovered mechanism of regulated cellular demise driven by excessive copper ion buildup (Cu^2+^). This process occurs when copper interacts with lipoylated mitochondrial proteins involved in respiration, triggering protein dysfunction and ultimately leading to the cell’s destruction. Unlike other forms of programmed cell death, this copper-dependent pathway creates unique proteotoxic conditions that prove fatal to the cell. Emerging evidence suggests that cuproptosis plays a critical role in tumor cell proliferation, metastasis, and chemoresistance [5]. Wang et al. developed a prognostic risk assessment model using cuproptosis-related long non-coding RNAs (lncRNAs), which accurately predicts the characteristic status of the tumor microenvironment [6]. Ferredoxin 1 (FDX1) catalyzes the reduction of Cu^2+^ to Cu^+^, the active form that promotes lipoylated protein aggregation and triggers cuproptosis. Previous studies have shown that FDX1 is highly expressed in glioma, where its primary function involves regulating the lipidation of tumor proteins and copper metabolism, significantly impacting the clinical prognosis of glioma patients [7]. Notably, the methylation modification of *FDX1* may drive the malignant progression of glioma. Mechanistic studies have revealed that cellular myelocytomatosis viral oncogene homolog (C-MYC), as a key regulator, strongly enhances glioma cell growth and spread via the YTH domain-containing family protein 1 (YTHDF1)-regulated FDX1 methylation mechanism. This process may be closely associated with the disruption of copper ion homeostasis in mitochondria [8]. Research on cuproptosis in glioma remains limited, and its precise mechanism of action is still unclear.

Existing studies have shown that postoperative chemotherapy is one of the most effective treatment strategies for managing GBM [9]. Temozolomide (TMZ) is a second-generation oral alkylating agent, and due to its ability to cross the blood–brain barrier, it is currently considered the first-line treatment for glioma [10]. The use of TMZ has significantly improved the median survival of patients; however, its efficacy often diminishes over time due to reduced sensitivity, ultimately leading to chemotherapy resistance [11]. Currently, overcoming TMZ chemoresistance is still a major clinical challenge in the treatment of GBM. Hence, delving into tactics to enhance the responsiveness of TMZ to chemotherapy is paramount. Significantly, cuproptosis has emerged as a pivotal factor in both tumor advancement and resistance to chemotherapy, including within glioma cells. Given that glioma cells usually exhibit dysregulation of copper homeostasis, we hypothesized that targeting copper metabolism might provide a promising strategy to overcome the resistance of glioma cells to TMZ. Understanding the relationship between TMZ sensitivity and copper death could uncover new therapeutic avenues for overcoming TMZ resistance. By inducing copper accumulation and promoting cuproptosis, it may be possible to enhance the therapeutic efficacy of TMZ in resistant glioma cells, thus improving patient outcomes. Therefore, exploring the synergistic effects of copper ions and TMZ in inducing cell death, particularly through the modulation of copper metabolism, is critical for identifying novel strategies to combat TMZ resistance in GBM.

The tripartite motif (TRIM) protein family includes more than 70 members, defined by a conserved N-terminal tripartite motif and a diverse C-terminal functional domain. Studies have shown that, as an important member of the E3 ubiquitin ligase family, most TRIM proteins regulate various cellular signaling pathways, including those involved in tumorigenesis, by modulating the ubiquitination of multiple substrate proteins [12]. Studies have found that Tripartite Motif-Containing 46 (TRIM46) mediates the ubiquitination of PH Domain and Leucine Rich Repeat Protein Phosphatase 2 (PHLPP2) via its PH domain, thereby activating the AKT/HK2 signaling pathway and ultimately promoting chemoresistance in lung cancer cells [13]. Liang et al. discovered that TRIM15 promotes the degradation of the Keap1–Nrf2 complex, thereby activating the oxidative stress response pathway and driving the malignant progression of non-small cell lung cancer [14]. TRIM14 may promote the malignant progression of glioma through dual molecular mechanisms: on one hand, by upregulating Zinc Finger E-box Binding Homeobox 2 (ZEB2) expression to induce epithelial–mesenchymal transition (EMT), thereby enhancing tumor cell invasiveness; on the other hand, by stabilizing Disheveled Segment Polarity Protein 2 (Dvl2) protein to activate the Wnt/β-catenin signaling pathway, ultimately contributing to the development of chemoresistance [15]. However, the precise molecular mechanisms by which TRIM14 functions in glioma remain unclear and warrant further investigation.

This study first reveals the possible mechanism by which TMZ and copper ions induce cuproptosis through targeting the TRIM14–ATP7A axis and emphasizes the potential value of TRIM14 in the prognosis evaluation and treatment of glioma.

## 2. Materials and Methods

### 2.1. Data Acquisition and Clinical Sample Collection

Comprehensive genomic and clinical data for patients diagnosed with glioblastoma (GBM) and low-grade glioma (LGG) were obtained from two major repositories: The Cancer Genome Atlas (TCGA, accessible at https://portal.gdc.cancer.gov/ (accessed on 8 April 2025)) and the Chinese Glioma Genome Atlas (CGGA, available through http://www.cgga.org.cn/ (accessed on 8 April 2025)). These datasets served as the foundation for our analysis. In total, 617 GBM and 516 LGG cases from TCGA, along with 693 glioma cases from CGGA, were analyzed in this study. In addition, clinical tumor specimens from patients with histologically confirmed GBM were collected at the First Affiliated Hospital of Ningbo University, with written informed consent obtained from all participants. All experimental procedures were conducted under protocols approved by the Ethics Committee of the First Affiliated Hospital of Ningbo University (Approval No. 2024-072A).

### 2.2. Bioinformatics Analysis

Differentially expressed genes (DEGs) were determined using the R package version of 4.4.2 “limma”, applying a threshold of |log2 fold change| > 1 together with an adjusted *p*-value < 0.05. To investigate the biological significance of the findings, we conducted functional enrichment analyses using the “clusterProfiler” package. This included Gene Ontology (GO) annotation and Kyoto Encyclopedia of Genes and Genomes (KEGG) pathway mapping. Additionally, we leveraged Gene Set Enrichment Analysis (GSEA) to identify key biological pathways correlated with TRIM14 expression patterns. Survival analyses were performed using the R packages “survival” and “survminer”. Kaplan–Meier (K–M) curves were constructed, and group differences were assessed via the log-rank test. Hazard ratios (HRs) with 95% confidence intervals (CIs) were estimated by means of Cox proportional hazards regression. A *p*-value less than 0.05 was considered statistically significant.

### 2.3. Cell Culture

The T98G and U251 human glioma cell lines were obtained from the Chinese Academy of Sciences’ Cell Bank in Shanghai. All experimental protocols followed the ethical guidelines approved by Ningbo First Hospital’s Human Ethics Committee in Ningbo, China. Cells were cultured in DMEM high-glucose medium supplemented with 10% FBS, NEAA, penicillin, and streptomycin (all from Gibco, Thermo Fisher Scientific, Waltham, MA, USA). They thrived under controlled conditions, where the temperature was a cozy 37 degrees Celsius, and the air was humid, with a gentle 5% CO_2_ blend in the 95% atmosphere. Temozolomide (TMZ; Sigma-Aldrich, St. Louis, MO, USA) was dissolved in DMSO, with the final solvent concentration kept below 0.1%. Copper(II) chloride (CuCl_2_; Sigma-Aldrich from Germany) was dissolved in sterile PBS and diluted to the indicated working concentrations for treatment.

### 2.4. RNA Interference and Vectors

Three small interfering RNAs (siRNAs) targeting TRIM14 were purchased from GenePharma, China. The sequences for the siRNAs were as follows: si-TRIM14-1: Sense: CCGAGAAGCUCAAGGCUAA, Antisense: UUAGCCUUGAGCUUCUCGG; si-TRIM14-2: Sense: CAGAUUACUACUUGACGAA, Antisense: UUCGUCAAGUAGUAAUCUG; si-TRIM14-3: Sense: CGUGCAGAAACUCAGCCAA, Antisense: UUGGCUGAGUUUCUGCACG.

### 2.5. Western Blotting

SDS-PAGE gel electrophoresis was performed, and protein transfer was carried out using a transmembrane transfer apparatus (Bio-Rad Laboratories, Hercules, CA, USA). PVDF membranes (Bio-Rad Laboratories, Hercules, CA, USA) were blocked with 5% non-fat milk at room temperature for 2 h. Primary antibodies were applied according to the manufacturer’s instructions and incubated at 4 °C for 12 h. Primary antibodies included ATP7A (Affinity Biosciences, Cincinnati, OH, USA, 1:1000), ATP7B (ProteinTech Group, Chicago, IL, USA, 1:1000), TRIM14 (ProteinTech Group, Chicago, IL, USA, 1:500), β-actin (ProteinTech Group, Chicago, IL, USA, 1:20,000), FDX1 (ProteinTech Group, Chicago, IL, USA, 1:1000), PCNA (ProteinTech Group, Chicago, IL, USA, 1:5000), Vimentin (ProteinTech Group, Chicago, IL, USA, 1:20,000), E-cadherin (ProteinTech Group, Chicago, IL, USA, 1:20,000), p100 (ProteinTech Group, Chicago, IL, USA, 1:5000), p52 (Affinity Biosciences, Cincinnati, OH, USA, 1:1000), SLC31A1 (ProteinTech Group, Chicago, IL, USA, 1:5000), Lip-DLAT (Affinity Biosciences, Cincinnati, OH, USA, 1:1000), Lip-DLST (Affinity Biosciences, Cincinnati, OH, USA, 1:1000), and others. After being rinsed three times with Tris-buffered saline containing Tween-20 (TBST), the membrane was subsequently incubated with a secondary antibody (ProteinTech Group, Chicago, IL, USA, 1:1000 dilution) for 1 h at room temperature. The immunoreactive bands were visualized using an enhanced chemiluminescence (ECL) reagent (New Cell & Molecular Biotech Co., Ltd., Suzhou, China).

### 2.6. Transwell Assay

Transwell chambers and Matrigel (Corning Inc., Corning, NY, USA) were employed for the assay. The lower chamber was filled with 500 μL of culture medium supplemented with 10% fetal bovine serum (FBS), and 5 × 10^4^ cells in a serum-free environment were placed in the upper compartment. The cells were cultured at 37 °C in a 5% CO_2_ atmosphere with 95% air and 100% humidity for 24 h. After staining with 0.1% crystal violet (Sinopharm Chemical Reagent Co., Ltd., Shanghai, China), the invasion rate was determined by counting the number of cells that had migrated.

### 2.7. Clone Formation

Logarithmic phase cells were seeded at 1000 cells/well into six-well plates. After two weeks, the colonies were fixed with methanol for 30 min, followed by staining with 0.1% crystal violet (Beyotime Biotechnology, Shanghai, China). The colonies were then manually counted to evaluate colony formation.

### 2.8. Tumor Xenograft Model

Female BALB/c nude mice (4–6 weeks old) were obtained from VITON Lihua (Shanghai, China) and housed under specific pathogen-free (SPF) conditions. All animal experiments were conducted in accordance with protocols approved by the Ethics Committee of the First Affiliated Hospital of Ningbo University (Approval No. 2024-072A). For xenograft experiments, U251 cells (5 × 10^6^ suspended in 100 μL PBS) were subcutaneously injected into the right flanks of the mice. Once tumors reached an approximate volume of 100 mm^3^, the animals were randomly divided into two groups (*n* = 5 per group): (1) vehicle control and (2) TMZ plus CuCl_2_ treatment. Temozolomide (TMZ, 10 mg/kg) was administered orally every other day for a total of 24 days. Copper(II) chloride (CuCl_2_, 1 mg/kg) was also given orally on alternate days for 24 consecutive days. Throughout the study, we meticulously measured the tumor size every three days with a caliper. The volume was then calculated with the following equation: volume (mm^3^) = (length × width^2^) × 0.5. Upon reaching the end of the experiment, the mice were humanely euthanized, and their tumor tissues were harvested for further histological and molecular investigation.

### 2.9. CCK-8 Cell Viability Assay

To evaluate the viability of U251 and T98G glioma cells, we performed a CCK-8 assay (Biosharp Life Sciences, Hefei, China) following the standard protocol. Cells in their logarithmic growth phase were plated in 96-well plates at 5000 cells per well, suspended in 100 μL of complete growth medium, and cultured overnight at 37 °C in a humidified 5% CO_2_ atmosphere. The original medium was removed, and different concentrations of copper ions (0–6 μM), TMZ (0–200 μM), or combination treatments were added to the wells (100 μL per well). Each group included 6 replicates, with a blank control group (no cells, only medium) and a negative control group (untreated cells). After 24 h of treatment, 10 μL of CCK-8 reagent was added to each well, and the cells were incubated for an additional 2 h. The absorbance at 450 nm was measured using a microplate reader.

### 2.10. Immunofluorescence

The cells were immobilized using a 4% paraformaldehyde solution and then treated with 0.5% Triton X-100 to allow permeation. Following a blocking step with 1% bovine serum albumin (BSA), the specimens were exposed to the primary antibody and subsequently subjected to an Alexa Fluor-labeled secondary antibody provided by AntGene Biotechnology Co., Ltd., Wuhan, China. Fluorescence images were acquired with a fluorescence microscope, and representative results are presented.

### 2.11. Immunohistochemical Staining

Tumor tissues were fixed in 4% formaldehyde, embedded in paraffin, and cut into 4-μm sections for immunohistochemistry (IHC). Endogenous peroxidase activity was quenched with 3% hydrogen peroxide for 10 min, after which nonspecific binding was blocked using 5% bovine serum albumin at room temperature for 30 min. The sections were then incubated overnight at 4 °C with primary antibodies, including PCNA (1:100, ProteinTech Group, Chicago, IL, USA), FDX1 (1:150, ProteinTech Group, Chicago, IL, USA), and ATP7A (1:200, Affinity Biosciences, Cincinnati, OH, USA). Subsequently, the samples were exposed to HRP-conjugated secondary antibodies (ProteinTech Group, Chicago, IL, USA) and counterstained with hematoxylin.

### 2.12. Flow Cytometry

Cells were digested, washed twice with PBS, and centrifuged at 2000 rpm for 5 min. Approximately 3 × 10^5^ cells were then transferred into flow cytometry tubes. The cells were fixed with pre-chilled 4% paraformaldehyde for 10 min, permeabilized with 0.25% Triton X-100 for 15 min, and subsequently blocked with 5% bovine serum albumin (BSA) at room temperature for 30 min. For cell cycle detection, cells were stained with propidium iodide (PI) prior to flow cytometric analysis. For apoptosis assays, cells were labeled with PI and Annexin V-FITC (Roche Diagnostics GmbH, Mannheim, Germany) following the manufacturer’s protocol. Data acquisition and analysis were performed using FlowJo software (version 10).

### 2.13. Fluorescence Probe Assays

For the detection of intracellular Cu^+^ accumulation, we utilized the CS1 fluorescence probe, a specific marker for copper ions. The procedure was as follows: Glioma cells were treated with CuCl_2_ (specific concentrations) alone or in combination with TMZ for the designated periods. After the treatment, cells were incubated with the CS1 fluorescence probe at the recommended concentration for 30 min at 37 °C. Cells were washed twice with PBS to remove excess probe. The fluorescence intensity was measured using a fluorescence microscope (excitation: 480 nm, emission: 510 nm). The intensity of the fluorescence signal was used to quantify the intracellular copper ion accumulation. The fluorescence signal was analyzed and compared between the treatment groups, with increased fluorescence indicating higher intracellular Cu^+^ accumulation.

### 2.14. Statistical Analysis

We ran our statistical analysis through SPSS 20.0 to crunch the numbers. All data points were reported as averages with their standard deviations (x ± s). When comparing just two groups, we employed independent sample *t*-tests, while for multiple group comparisons, we utilized one-way ANOVA supplemented by Tukey’s post hoc analysis to pinpoint any significant differences. A *p*-value < 0.05 was considered statistically significant. * *p* < 0.05, ** *p* < 0.01, *** *p* < 0.001. Quantification of clinical Western blotwas analyzed by densitometry using ImageJ software version of 1.53t, and statistical significance was determined using Student’s *t*-test (*n* = 2 pairs of clinical samples).

## 3. Results

### 3.1. CuCl_2_ Enhances the Cytotoxicity of TMZ on Glioblastoma Cells and Inhibits Proliferation and Invasion

**Figure 1:**
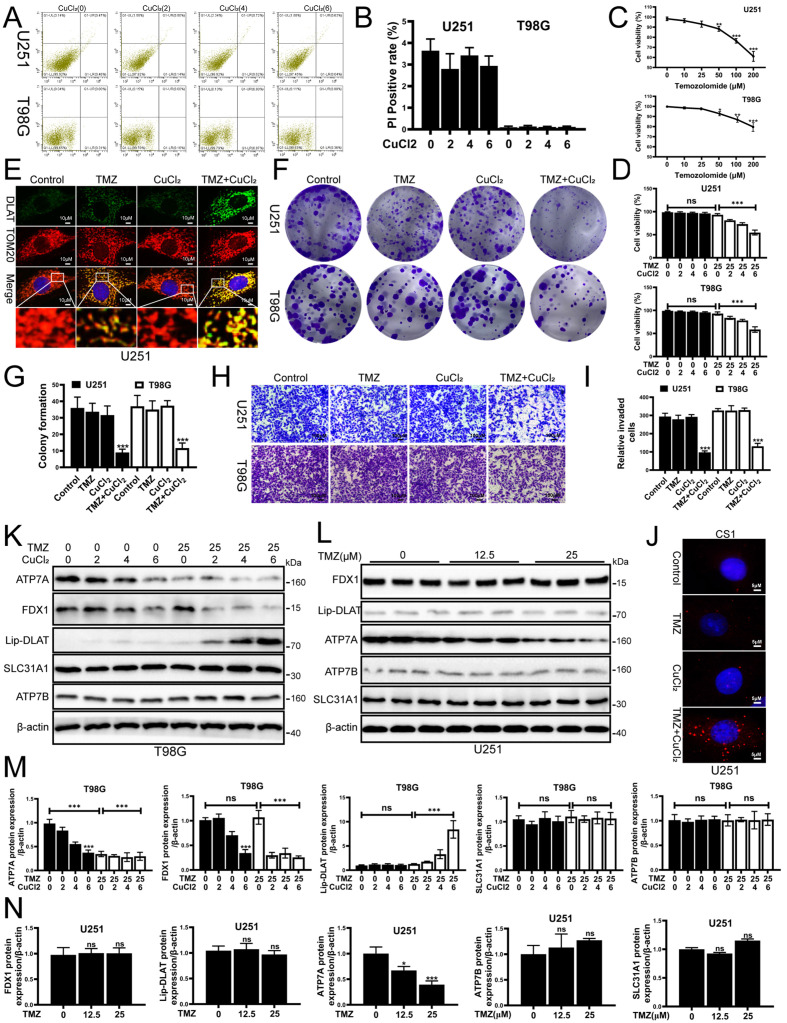
CuCl_2_ Enhances TMZ Cytotoxicity on Glioblastoma Cells and Inhibits Proliferation and Invasion. (**A**) Flow cytometry analysis of apoptosis in U251 (upper row) and T98G (lower row) cells treated with different concentrations of CuCl_2_ (0–6 μM). (**B**) Quantification of PI-positive cells in U251 and T98G cells after CuCl_2_ (0–6 μM) treatment, showing that CuCl_2_ alone has low toxicity (*n* = 3). (**C**) Dose–response curves of U251 and T98G cell viability after treatment with different concentrations of TMZ (0–200 μM) (*n* = 3). (**D**) Cell viability of U251 and T98G cells treated with TMZ (25 μM) alone or in combination with different concentrations of CuCl_2_ (0–6 μM) (*n* = 3). (**E**) Immunofluorescence staining of U251 cells after different treatments. Green represents DLAT, red represents the mitochondrial marker TOM20, and yellow indicates colocalization of both (63×). (**F**,**G**) Representative images and quantitative analysis of clonogenic assays for U251 and T98G cells under different treatments (*n* = 3). (**H**,**I**) Representative images and quantification of Transwell invasion assays showing that the invasion ability significantly decreased in the combined treatment group (*n* = 3) (20×). (**J**) CS1 fluorescence probe assay to detect Cu^+^ levels in U251 and T98G cells, showing significant Cu^+^ accumulation in the combined treatment group (100×). (**K**,**M**) Western blot and grayscale analysis showing the expression of copper transport and cuproptosis-related proteins (ATP7A, FDX1, Lipoylated-DLAT, SLC31A1, ATP7B) in T98G cells after treatment with TMZ (0, 25 μM) and CuCl_2_ (0–6 μM) (*n* = 3). (**L**,**N**) Western blot and grayscale analysis showing the expression of the same proteins in U251 cells treated with different concentrations of TMZ (0, 12.5, 25 μM) (*n* = 3) (* *p* < 0.05, ** *p* < 0.01, *** *p* < 0.001, ns: not significant).

Emerging research highlights the critical influence of cuproptosis on cancer progression, demonstrating its impact on tumor growth, spread, and treatment resistance. Lu et al. found that FDX1, as a cuproptosis-linked gene, is closely related to the immune infiltration of glioma, shedding new light on potential therapeutic targets [7]. Gao et al. found that DLAT, as a cuproptosis-linked gene, was associated with severe clinical features and poor immune infiltration in glioma [16]. To investigate whether copper ions (Cu) could enhance the death of glioma cells, we treated U251 and T98 glioma cells with copper ions. Flow cytometry results showed that CuCl_2_ (0–6 μM) alone did not significantly induce apoptosis in both U251 and T98G cell lines, and cell viability remained stable (Figure 1A,B). Literature has reported that iron ions can promote oxidative stress-induced death. Therefore, we are interested in whether copper ions can enhance glioma cell sensitivity to TMZ chemotherapy. Subsequently, we treated glioma cells with different concentrations of temozolomide (TMZ). The results showed that TMZ had no significant effect on the viability of U251 and T98G cells within a certain concentration range (Figure 1C). In the subsequent experiments, we used a low, non-cytotoxic concentration of TMZ to model TMZ-insensitive conditions in glioma cells. Interestingly, adding a low dose of CuCl_2_ to TMZ significantly enhanced TMZ’s cytotoxicity, resulting in a notable decrease in cell viability (Figure 1D), a significant reduction in clonogenic ability (Figure 1F,G), and a marked inhibition of migration and invasion (Figure 1H,I). Immunofluorescence detection showed that the Dihydrolipoamide S-acetyltransferase (DLAT) and Translocase of Outer Mitochondrial Membrane 20 (TOM20) colocalization signal was enhanced in the TMZ+CuCl_2_ treatment group, suggesting that mitochondrial-associated lipoylation processes were altered (Figure 1E). Meanwhile, CS1 probe results showed that TMZ+CuCl_2_ treatment led to a significant accumulation of Cu^+^ inside the cells (Figure 1J). At the molecular level, Western blot results indicated that in T98G cells, ATP7A was significantly downregulated as the CuCl_2_ concentration increased, and FDX1 showed a downward trend, while the expression of SLC31A1 and ATP7B did not show significant changes (Figure 1K,M). Comparatively, solitary TMZ had no significant impact on the proteins’ expression within U251 cells (Figure 1L,N). In summary, the in vitro experimental results suggest that exogenous copper ions are minimally toxic but significantly enhance the inhibitory effect of TMZ on glioblastoma cells. This effect is accompanied by intracellular Cu^+^ accumulation and alterations in copper transport/cuproptosis pathway proteins.

### 3.2. TMZ+CuCl_2_ Inhibits Glioblastoma Growth in Vivo and Reverses EMT, Accompanied by Changes in Copper Homeostasis-Related Protein Expression

**Figure 2:**
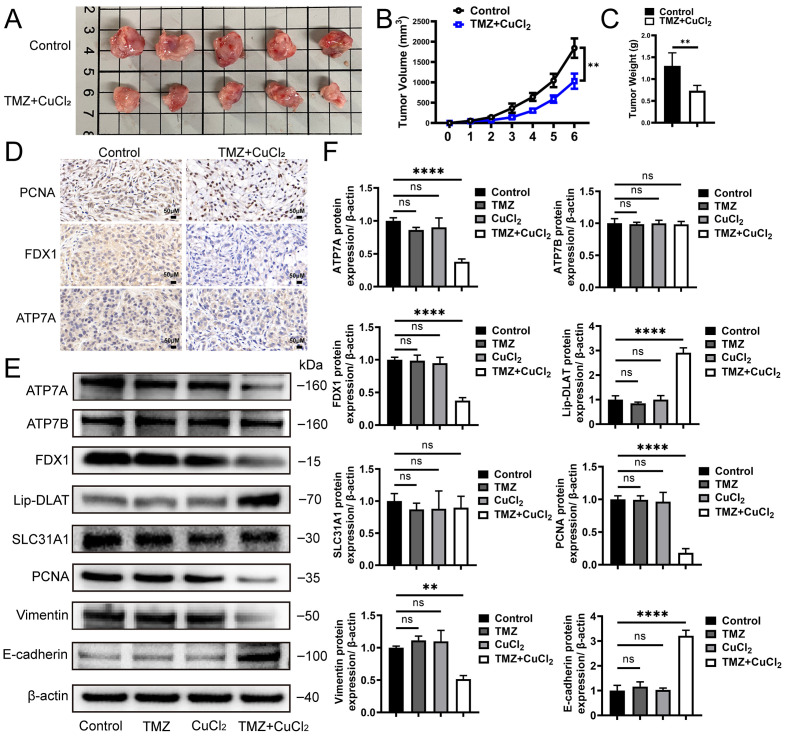
TMZ+CuCl_2_ Combined Treatment Inhibits Tumor Growth in Mice and Alters Copper Homeostasis and EMT-Related Protein Expression. (**A**) Representative images of subcutaneous xenograft tumors in nude mice after different treatments. The tumor volume in the control group is larger, while the combined treatment group shows significantly smaller tumors. (**B**) Dynamic changes in tumor volume for each group, showing a significant inhibition of tumor growth in the combined treatment group (*n* = 5). (**C**) Statistical analysis of tumor weight at the end of the experiment, with the combined treatment group showing significantly lower tumor weight compared to the control group (*n* = 5). (**D**) Representative images of immunohistochemical staining of xenograft tumors showing the expression levels of proliferation-related protein PCNA and copper death-related proteins FDX1 and ATP7A. In the combined treatment group, the proportion of positive cells for PCNA, FDX1, and ATP7A is reduced (20×). (**E**) Western blot analysis of copper transport and copper deposition-related proteins (ATP7A, FDX1, Lipoylated-DLAT, SLC31A1, ATP7B) and proliferation/emt-related proteins (PCNA, vimentin, E-cadherin) in the control group, only TMZ group, only CuCl_2_ group and combination treatment group. (**F**) Grayscale analysis of protein expression showing significant downregulation of ATP7A, FDX1, PCNA, and Vimentin, and upregulation of E-cadherin in the combined treatment group (*n* = 3) (** *p* < 0.01, **** *p* < 0.0001, ns: not significant).

In the in vivo experiment, we further verified the sensitizing effect of CuCl_2_ and the antitumor effect of TMZ. After xenotransplantation of U251 cells into balb/c nude mice, we evaluated the in vivo function of tmz+cu. We orally administered TMZ (10 mg/kg) and CuCl_2_ (1 mg/kg) to mice. The concentration of TMZ showed no significant therapeutic effect, and the safe dose of CuCl_2_. The results showed that the tumor volume of the combined treatment group was significantly smaller than that of the control group (Figure 2A). The tumor volume curve showed that the tumor growth was significantly slowed down in the treatment group (Figure 2B), and the final tumor weight was also significantly reduced (Figure 2C). Immunohistochemical analysis showed that the expression level of proliferation-related proteins in tumor tissues of the combined treatment group decreased, while the distribution of apoptosis markers and copper death-related proteins changed. Compared with the control group, TMZ+CuCl_2_ significantly reduced the intensity of PCNA, fdx1 and ATP7A immunostaining. (Figure 2D). To further verify the mechanism observed in the in vitro experiments, we examined the expression changes in related proteins in each group. Western blot analysis showed that there was no significant difference in the expression of each protein between the TMZ-only group and the Cu-only group compared with the control group. Compared with the control group, the expression of ATP7A in the TMZ+CuCl_2_ combined treatment group was significantly downregulated, and FDX1 was also downregulated, while SLC31A1 and ATP7B had no significant changes. Compared with the control group, the lipoylated-DLAT signal was significantly changed (Figure 2E,F). These results suggest that copper ions similarly trigger the remodeling of copper transport and copper exophthalmos-related pathways in vivo. Meanwhile, the combined treatment significantly affected the expression of tumor phenotype-related proteins: the level of PCNA decreased, indicating that tumor cell proliferation was inhibited; Vimentin was downregulated, while E-cadherin was upregulated, indicating that the EMT process was inhibited (Figure 2E,F). These results are consistent with the reduced clonogenic ability and reduced invasion and migration observed in in vitro experiments.

### 3.3. Transcriptome Sequencing and WB Validation Reveal That TMZ+CuCl_2_ Combined Treatment Inhibits the NF-κB Pathway by Downregulating TRIM14 and Reshapes Metabolic Homeostasis

**Figure 3:**
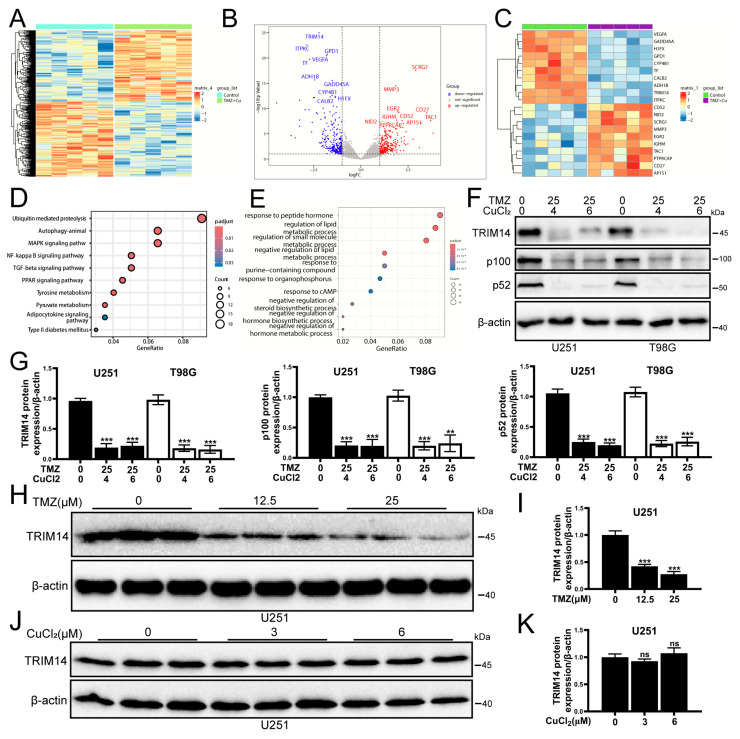
Transcriptome Sequencing and WB Validation Reveal That TMZ+CuCl_2_ Combined Treatment Inhibits TRIM14 and the NF-κB2 Pathway and Reshapes Metabolic Homeostasis. (**A**) Volcano plot showing the differentially expressed genes between the combined treatment group and the control group, with upregulated genes in red and downregulated genes in blue. (**B**) Heatmap showing the clustering of differential gene expression between the combined treatment group and the control group, indicating a significant separation in overall transcriptional profiles. (**C**) Statistical analysis of differential gene expression. (**D**) KEGG pathway enrichment analysis showing that differentially expressed genes are significantly enriched in pathways related to ubiquitin-mediated proteolysis, autophagy, MAPK/NF-κB, TGF-β, PPAR, and pyruvate metabolism. (**E**) GO biological process enrichment analysis showing that differentially expressed genes are mainly enriched in processes related to hormone and lipid metabolism regulation, cAMP response, purine metabolism, and chemical stress response. (**F**) Western blot analysis showing the expression of TRIM14, p100, and p52 proteins in U251 and T98G cells treated with TMZ (25 μM) ± CuCl_2_ (0–6 μM). (**G**) Quantification of grayscale values showing significant downregulation of TRIM14, p100, and p52 in the combined treatment group (*n* = 3). (**H**,**I**) TRIM14 protein expression and quantification in U251 cells treated with different concentrations of TMZ (0, 12.5, 25 μM), showing a dose-dependent decrease (*n* = 3). (**J**,**K**) TRIM14 protein expression and quantification in U251 cells treated with different concentrations of CuCl_2_ (0, 3, 6 μM), with no significant differences. (*n* = 3) (** *p* < 0.01, *** *p* < 0.001, ns: not significant).

To further explore the molecular mechanisms of TMZ+CuCl_2_ combined treatment, we performed transcriptome sequencing analysis on U251 cells. Differential gene analysis (Figure 3A) revealed multiple upregulated and downregulated genes in the combined treatment versus the control. Among the upregulated genes, the top ten are SCRG1, MMP3, EGR2, CD27, IGHM, CD52, TAC1, NID2, AP1S1, and PTPRCAP. The top ten downregulated genes are TRIM14, ITPKC, GPD1, VEGFA, TF, ADH1B, GADD45A, CYP4B1, H1FX, and CALB2. Heatmap clustering (Figure 3B,C) further suggested that the two groups of samples could be clearly distinguished in the overall transcriptomic profile, indicating that the combined treatment significantly reshapes the gene expression pattern. Analysis of the differentially expressed genes revealed distinct biological patterns associated with TMZ+CuCl_2_ treatment. The upregulated gene set was enriched for signatures related to immune cell activation and infiltration, extracellular matrix (ECM) remodeling, and inflammatory response pathways, alongside genes involved in cellular stress adaptation and modulation of cell adhesion. These transcriptional changes indicate a shift toward an immune-responsive and microenvironment-remodeling state under combined treatment. Conversely, the downregulated genes highlighted several pathways that may contribute to enhanced therapeutic vulnerability. Notably, suppression of TRIM14, a key stabilizer of ATP7A, suggests inhibition of the TRIM14–ATP7A copper efflux axis, potentially promoting intracellular copper accumulation. Additional downregulated signatures pointed to disruption of metabolic and redox homeostasis, marked reduction in angiogenic signaling (e.g., VEGFA), and attenuation of DNA damage response mechanisms (e.g., GADD45A). Together, these alterations reflect an impaired adaptive capacity of tumor cells when exposed to TMZ in the presence of elevated copper levels. GO enrichment analysis (Figure 3E) showed that the differentially expressed genes were predominantly enriched in biological processes associated with hormone and lipid metabolic regulation, redox homeostasis, purine metabolism, cAMP signaling, and cellular responses to chemical stress. Importantly, many of these processes are tightly linked to mitochondrial function and oxidative vulnerability, suggesting that the combined treatment substantially perturbs cellular metabolic equilibrium. KEGG pathway enrichment analysis (Figure 3D) further highlighted that the differentially expressed genes were significantly enriched in ubiquitin-mediated proteolysis, autophagy, and MAPK/NF-κB signaling, alongside TGF-β, PPAR signaling, and pyruvate/tyrosine metabolism. Notably, several of these pathways—particularly ubiquitin–proteasome regulation, autophagy, and MAPK/NF-κB inflammatory signaling—represent key nodes involved in copper homeostasis, protein quality control, and stress adaptation. Together, these findings indicate that TMZ+CuCl_2_ co-treatment exerts a coordinated, multi-level impact on tumor cells, prominently disrupting protein degradation pathways, inflammatory and stress response signaling, and mitochondrial and lipid metabolism, all of which may collectively sensitize glioblastoma cells to copper-induced cytotoxicity. To validate the transcriptome results, we examined the expression of TRIM14 and NF-κB2 pathway proteins (p100/p52). The results showed (Figure 3F,G) that under TMZ+CuCl_2_ conditions, TRIM14, p100, and p52 were significantly downregulated in both cell lines. Dose–response experiments further showed (Figure 3H,I) that TMZ alone could dose-dependently downregulate TRIM14, whereas CuCl_2_ alone had no significant effect on TRIM14 (Figure 3J,K). This indicates that TMZ is the main driver of TRIM14 downregulation, while CuCl_2_ synergistically enhances this inhibitory effect.

### 3.4. Overexpression of TRIM14 Is Associated with Poor Survival in Glioma Patients: Analysis of TCGA and CGGA Databases

**Figure 4:**
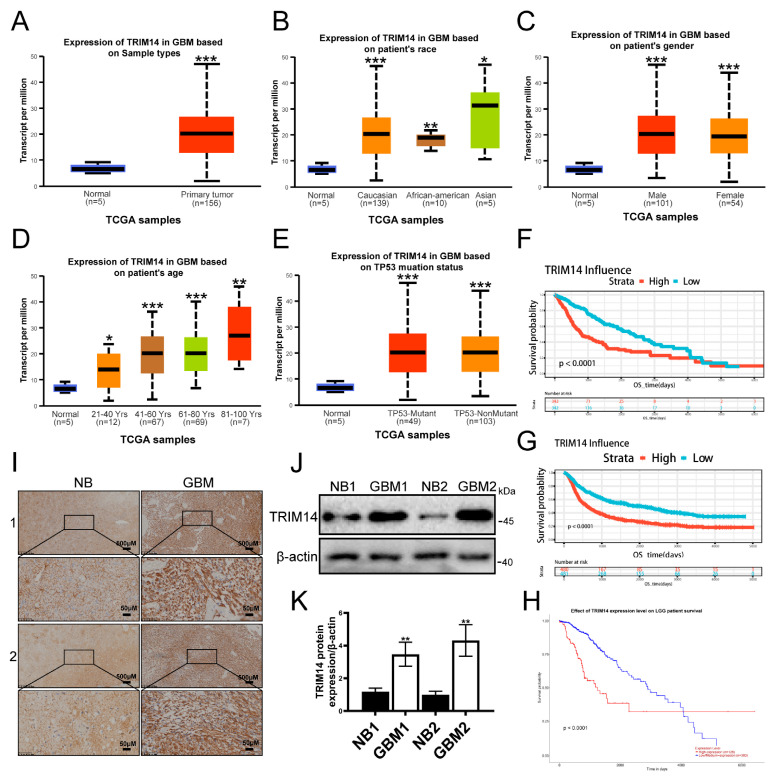
High Expression of TRIM14 in Gliomas Correlates with Poor Prognosis. (**A**) Comparison of TRIM14 expression between normal brain tissue and GBM primary tumors in the TCGA cohort. (**B**,**C**) TRIM14 expression distribution stratified by race and gender, showing that tumor samples have higher expression than normal tissue. (**D**) TRIM14 expression stratified by age, showing an increase with age. (**E**) TRIM14 expression distribution in TP53-mutated and non-mutated GBM, both showing higher expression than normal tissue. (**F**,**G**) OS survival curves for high/low TRIM14 expression in GBM, showing significantly poorer survival in the high-expression group. (**H**) OS survival curve for high/low TRIM14 expression in LGG patients, with high expression indicating worse prognosis. (**I**) Representative immunohistochemical staining of TRIM14 in normal brain (NB) and GBM tissues, with stronger positivity in GBM (4×, 20×). (**J**) Western blot of TRIM14 protein expression in two pairs of normal brain and GBM tissues. (**K**) Grayscale quantification of Western blot results showing significantly higher TRIM14 expression in GBM compared to NB (*p* < 0.01) (*n* = 3) (* *p* < 0.05, ** *p* < 0.01, *** *p* < 0.001).

To assess the clinical relevance of TRIM14 in glioma, we first analyzed the TCGA database. The results showed that TRIM14 expression in primary GBM tumor samples was significantly higher than in normal brain tissue (*p* < 0.001) (Figure 4A). Further stratified analysis revealed that TRIM14 expression was elevated in tumor patients of different ethnicities and genders compared to normal tissue (*p* < 0.05, *p* < 0.01, *p* < 0.001) (Figure 4B,C), suggesting that this upregulation is widespread. Upon grouping by age, we found that TRIM14 was expressed at higher levels in older patients (*p* < 0.05, *p* < 0.01, *p* < 0.001) (Figure 4D). Additionally, TRIM14 expression was higher in both TP53-mutated and non-mutated samples compared to normal tissues (*p* < 0.001) (Figure 4E). Survival analysis revealed that patients with high TRIM14 expression had a significantly shorter overall survival. High TRIM14 expression was associated with worse prognosis in both GBM and LGG cohorts (Figure 4F–H). To further verify these results, we examined the protein levels of TRIM14 in clinical tissue samples. Immunohistochemical results showed that TRIM14-positive signals were significantly stronger in GBM tissues compared to normal brain tissue (Figure 4I). Western blot results also indicated that the TRIM14 protein level was significantly higher in GBM tissues compared to corresponding normal brain tissues (Figure 4J,K). In conclusion, TRIM14 is significantly upregulated in gliomas, and high expression is closely associated with poor prognosis in patients.

### 3.5. Overexpression of TRIM14 Reduces Cu^+^ Accumulation and Weakens the Antitumor Effect of TMZ+CuCl_2_

**Figure 5:**
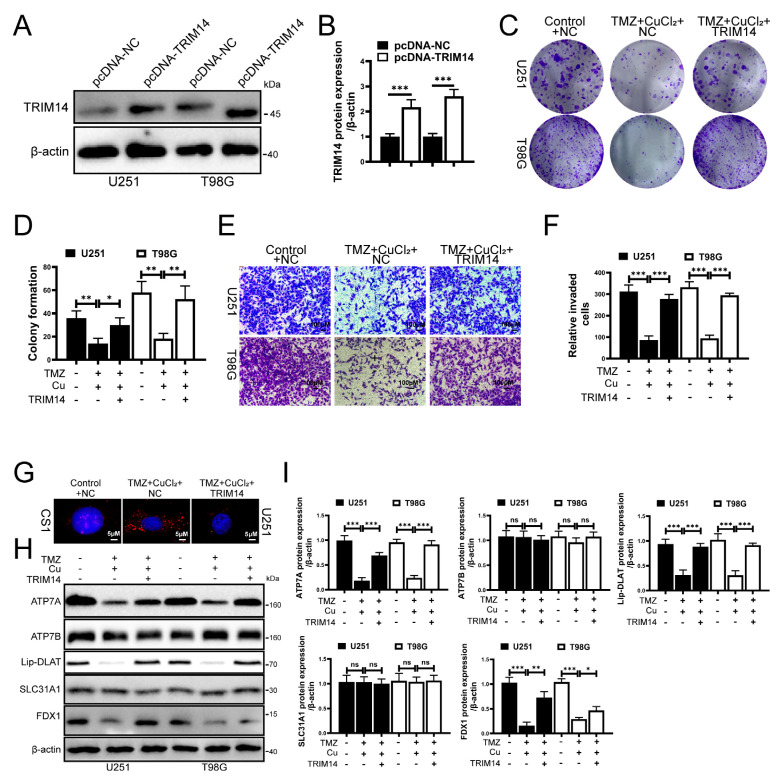
TRIM14 Overexpression Reduces Cu^+^ Accumulation and Weakens TMZ+CuCl_2_ Antitumor Effects. (**A**,**B**) Western blot validation of TRIM14 overexpression efficiency (*n* = 3). (**C**,**D**) Clonogenic assay: TRIM14 overexpression partially restores the inhibition of cell proliferation by TMZ+CuCl_2_ (*n* = 3). (**E**,**F**) Transwell invasion assay: TRIM14 overexpression significantly enhances cell invasion ability and weakens the inhibitory effect of TMZ+CuCl_2_ (*n* = 3) (10×). (**G**) CS1 fluorescence probe detection of Cu^+^ levels, showing significant Cu^+^ accumulation in the combined treatment group, while fluorescence signals are significantly reduced in the TRIM14 overexpression group (100×). (**H**,**I**) Western blot analysis of copper homeostasis and cuproptosis-related proteins, showing that TRIM14 overexpression reverses the downregulation of ATP7A, FDX1, and Lipoylated-DLAT, and the upregulation of SLC31A1, while restoring ATP7B expression (*n* = 3) (* *p* < 0.05, ** *p* < 0.01, *** *p* < 0.001, ns: not significant).

To elucidate the role of TRIM14 in the synergistic effect of TMZ+CuCl_2_, we constructed TRIM14-overexpressing cells and conducted functional and mechanistic validation. Western blot analysis showed that TRIM14 was significantly upregulated in the overexpression group, confirming the reliability of the model (Figure 5A,B). At the functional level, clonogenic assays showed that overexpression of TRIM14 partially restored the inhibition of cell clonogenic ability induced by TMZ+CuCl_2_ (Figure 5C,D). Transwell invasion assays showed that overexpression of TRIM14 significantly enhanced the cell membrane penetration ability, thereby weakening the anti-invasion effect of TMZ+CuCl_2_ (Figure 5E,F). CS1 fluorescence probe detection revealed that Cu^+^ signals were significantly enhanced in control cells under TMZ+CuCl_2_ treatment, whereas fluorescence signals in TRIM14 overexpressing cells were markedly reduced (Figure 5G). Further Western blot results showed that under TMZ+CuCl_2_ treatment, TRIM14 overexpression reversed the downregulation of ATP7A, FDX1, and Lipoylated-DLAT, weakened the upregulation of SLC31A1, and restored ATP7B expression (Figure 5H,I). In summary, overexpression of TRIM14 weakens the antitumor effect of TMZ+CuCl_2_ by reducing Cu^+^ accumulation and restoring copper homeostasis and the expression of cuproptosis pathway-related proteins.

### 3.6. Knockdown of TRIM14 Promotes TMZ+CuCl_2_-Induced Copper Ion Accumulation and Cuproptosis

**Figure 6:**
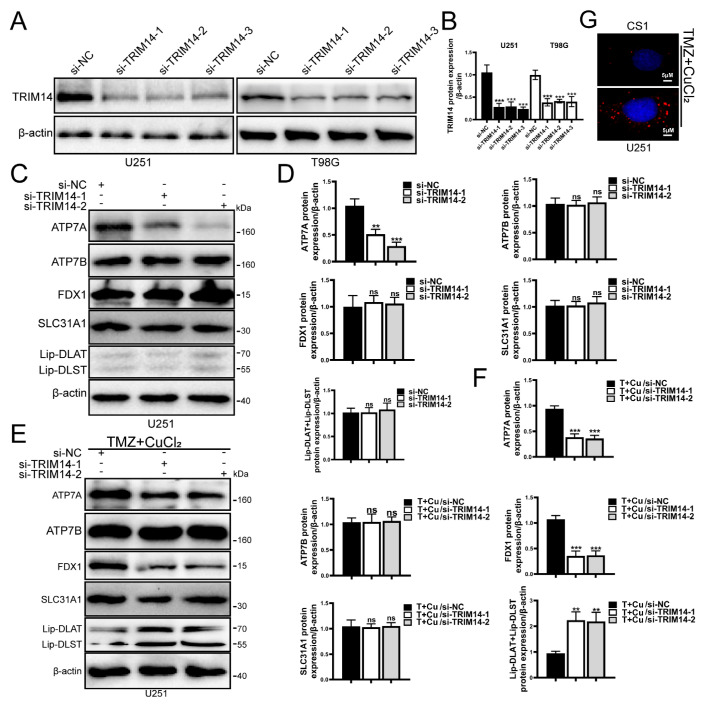
TRIM14 knockdown promotes TMZ+CuCl_2_-induced copper ion accumulation and cuproptosis. (**A**,**B**) Western blot validation of TRIM14 knockdown efficiency, showing a significant reduction in TRIM14 expression in the sh-TRIM14 group (*n* = 3). (**C**–**F**) Western blot analysis of copper homeostasis- and cuproptosis-related proteins. TRIM14 knockdown resulted in downregulation of ATP7A, while ATP7B and SLC31A1 showed no significant changes. Under TMZ+CuCl_2_ treatment, FDX1 expression was decreased, whereas lipoylated-DLAT and lipoylated-DLST were upregulated, indicating enhanced mitochondrial lipoylated protein aggregation and amplified cuproptosis (*n* = 3). (**G**) CS1 fluorescence probe detection of intracellular Cu^+^ levels, showing that TMZ+CuCl_2_ treatment induced Cu^+^ accumulation, which was further elevated in the TRIM14 knockdown group (100×) (** *p* < 0.01, *** *p* < 0.001, ns: not significant).

To further clarify the role of TRIM14 in TMZ+CuCl_2_-induced cuproptosis, we knocked down TRIM14 in glioma cells. First, we assessed the knockdown efficiency of TRIM14, and Western blot analysis showed that TRIM14 was significantly reduced in the knockdown group, confirming the successful construction of the model (Figure 6A,B). CS1 fluorescence probe detection showed that TMZ+CuCl_2_ treatment induced Cu^+^ accumulation within the cells, and in the TRIM14 knockdown background, CS1 fluorescence signals were significantly enhanced, suggesting that TRIM14 deficiency further promoted Cu^+^ accumulation in the cells (Figure 6G). Further Western blot analysis of copper homeostasis and cuproptosis key proteins showed that ATP7A was significantly downregulated in the TRIM14 knockdown group, indicating a weakened copper efflux capacity in the cells. There were no significant differences in ATP7B and SLC31A1 expression between the control and TRIM14 knockdown groups. Regardless of TMZ+CuCl_2_ treatment, ATP7A expression was downregulated; under TMZ+CuCl_2_ treatment, FDX1 expression decreased, and lipoylated-DLAT and lipoylated-DLST expression significantly increased, while ATP7B and SLC31A1 showed no significant differences. FDX1 was significantly downregulated under TMZ+CuCl_2_ treatment, suggesting that key initiators of cuproptosis were suppressed. Lipoylated-DLAT and lipoylated-DLST were significantly upregulated under TMZ+CuCl_2_ treatment, indicating enhanced accumulation of mitochondrial lipoylated proteins, a characteristic molecular feature of cuproptosis (Figure 6C–F).

### 3.7. High Expression of ATP7A in Glioma Is Associated with Poor Prognosis and Potential Interaction with TRIM14

**Figure 7:**
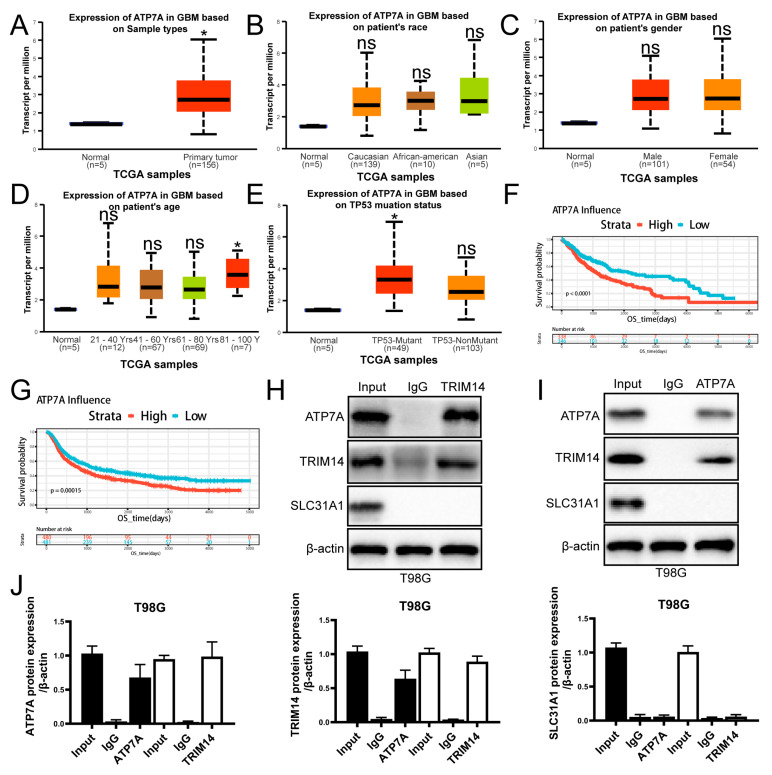
High Expression of ATP7A in Gliomas Correlates with Poor Prognosis and Interacts with TRIM14. (**A**) Comparison of ATP7A expression between normal brain tissue and GBM primary tumors in the TCGA cohort. (**B**,**C**) ATP7A expression distribution stratified by race and gender. (**D**) ATP7A expression stratified by age. (**E**) ATP7A expression distribution in TP53-mutated and non-mutated GBM, both showing higher expression than normal tissue. (**F**,**G**) OS survival curves for high/low ATP7A expression in GBM, showing significantly poorer survival in the high-expression group (*p* < 0.0001). (**H**–**J**) Co-IP analysis showing that TRIM14 interacts with ATP7A (*n* = 3) (* *p* < 0.05, ns: not significant).

**Figure 8:**
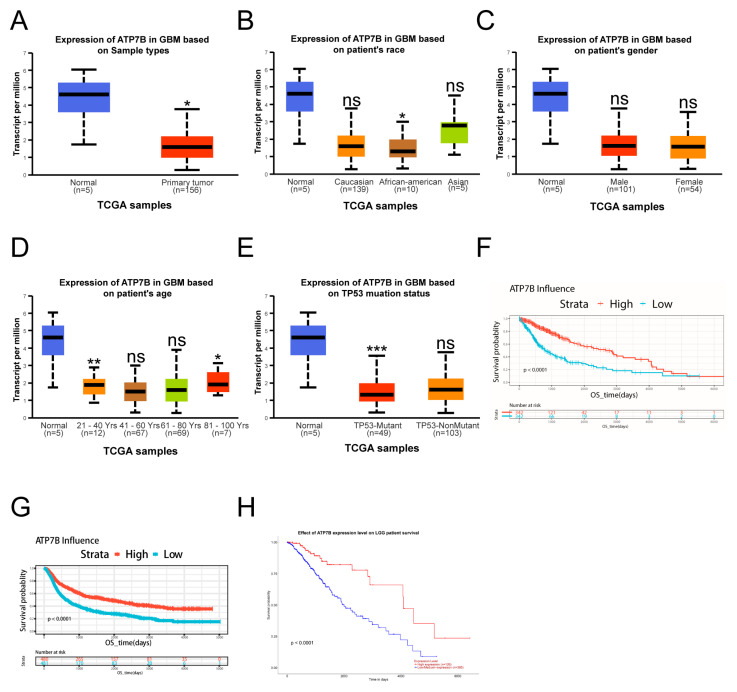
Low Expression of ATP7B in Glioma Patients. (**A**) ATP7B expression in GBM samples. (**B**) ATP7B expression distribution based on patient race. (**C**) ATP7B expression distribution based on patient gender. (**D**) ATP7A expression in GBM based on patient age. (**E**) ATP7A expression in GBM based on patient TP53 mutation status. (**F**–**H**) Kaplan-Meier (KM) curves showing prognosis of patients with high or low ATP7B expression in the TCGA-GBM, CGGA, and TCGA-LGG databases (* *p* < 0.05, ** *p* < 0.01, *** *p* < 0.001, ns: not significant).

To further explore the clinical significance of ATP7A in glioma and its relationship with TRIM14, we first conducted an analysis using public databases. Results from the TCGA dataset show that ATP7A expression is significantly higher in glioma tissues compared to normal brain tissue (Figure 7A). However, ATP7A expression did not show significant differences among GBM patients of different races or genders (Figure 7B,C). Among GBM patients of different ages, no significant differences were observed compared to normal controls, except for patients older than 80 years, who showed a significant increase in ATP7A expression (*p* < 0.05) (Figure 7D). In TP53-mutant and non-mutant samples, ATP7A expression in TP53-mutant cases was significantly higher than that in normal tissue (*p* < 0.05) (Figure 7E). Survival analysis further revealed that patients with high ATP7A expression have significantly shorter overall survival, indicating that high ATP7A expression is closely associated with poor prognosis, which is also applicable to LGG patients (Figure 7F,G). On the mechanistic level, we investigated the interaction between TRIM14 and ATP7A through co-immunoprecipitation (Co-IP) experiments. The Co-IP results suggest that TRIM14 may form a complex with ATP7A, supporting the potential interaction between the two (Figure 7H–J). Western blotting results showed that overexpression of TRIM14 upregulated ATP7A (Figure 5H), while TRIM14 knockdown downregulated ATP7A (Figure 6C), suggesting that TRIM14 may influence ATP7A levels through regulatory mechanisms. In contrast, ATP7B expression in glioma tissues was significantly lower than in normal brain tissues (*p* < 0.05) (Figure 8A). Among different race groups, only African American patients showed a significant difference compared with the normal controls (*p* < 0.05), while no significant differences were observed in other groups (Figure 8B). No significant difference was detected between male and female patients (Figure 8C). Across different age groups, GBM patients aged 21–40 years and those older than 80 years showed significant differences compared with normal controls (*p* < 0.01, *p* < 0.05) (Figure 8D). In TP53-mutant samples, ATP7B expression was significantly lower than in normal tissue (*p* < 0.001) (Figure 8E). Survival analysis showed that patients with low ATP7B expression had significantly shorter overall survival in both GBM and LGG cohorts, indicating that the loss or downregulation of ATP7B is closely associated with poor prognosis (Figure 8F–H). In conclusion, the results suggest that the expression patterns and clinical significance of ATP7A and ATP7B in glioma differ: high ATP7A expression is associated with poor prognosis, while ATP7B downregulation also predicts poor clinical outcomes. Further mechanistic studies suggest that TRIM14 may regulate ATP7A protein levels through interaction, highlighting the potential role of the TRIM14–ATP7A axis in copper homeostasis and glioma progression.

## 4. Discussion

Glioblastoma multiforme (GBM) is a highly heterogeneous and invasive primary brain tumor. The median survival time of GBM patients is less than 15 months [2]. Currently, the clinical treatment options for glioblastoma are very limited, with temozolomide (TMZ) still being the only approved standard chemotherapy regimen for first-line treatment. Although TMZ shows clinical benefits in the initial treatment of GBM patients, prolonged use may lead to reduced responsiveness of tumor cells and ultimately induce resistance [17]. Therefore, exploring novel sensitization strategies to overcome therapeutic bottlenecks is a core direction in glioma research.

In recent years, copper-induced cell death (cuproptosis), as a novel form of programmed cell death, has attracted significant attention in the field of cancer therapy [5]. Studies have found that the copper death-related gene FDX1 is abnormally expressed in glioma tissues, suggesting that copper metabolism imbalance may be involved in glioma progression [7]. The core mechanism of cuproptosis relies on the accumulation of toxic monovalent copper ions (Cu^+^) in the mitochondria, which directly bind to acetylated TCA cycle enzymes (such as DLAT), leading to toxic protein aggregation and mitochondrial dysfunction [5]. As cuproptosis has been established as a novel form of cell death, targeting this mechanism in tumor cells is considered to have potential strategic value in overcoming tumor resistance. Studies have shown that Elesclomol–Cu in prostate cancer can enhance cellular response to docetaxel by activating copper accumulation dependent on the DLAT/mTOR pathway (which occurs both in vitro and in vivo) [18]. A study pointed out that disulfiram–Cu can overcome drug resistance to bortezomib and cytarabine in Down syndrome-related acute myeloid leukemia cell lines, potentially involving the induction of apoptosis and inhibition of proteasome function recovery [19]. This study first discovered that TMZ+CuCl_2_ co-treatment enhances the chemotherapeutic sensitivity of TMZ in GBM cells by inducing cuproptosis, which may provide a new strategy for overcoming TMZ resistance.

Although it is widely believed that increased intracellular copper ions can induce cell death, our study found that copper ions within a certain concentration range do not significantly inhibit GBM cell activity. The absorption, distribution, transport, and elimination of copper ions in the human body are finely regulated, maintaining copper metabolism homeostasis, which plays an important role in normal physiological functions [20]. Hamza et al. found that when Cu^+^ accumulates excessively in the cell, it can enter the trans-Golgi network (TGN) and then be transported out of the cell via vesicular trafficking [21]. The increase in intracellular Cu content can also drive the relocation of ATP7A and ATPase Copper-Transporting Beta (ATP7B) within the TGN region, thereby enhancing the process of copper ion export [22]. Therefore, low concentrations of copper ions can still be exported from the cell through a compensatory mechanism. While our study primarily focuses on the role of copper ions in enhancing the sensitivity of glioblastoma cells to temozolomide via cuproptosis, it is essential to consider the potential risk of copper overload in vivo, particularly when using copper-based therapies. Excessive copper accumulation can lead to toxicity, with the potential to disrupt normal cellular functions and contribute to systemic copper overload. Copper homeostasis in the body is tightly regulated through transporters such as ATP7A and ATP7B, which mediate copper efflux and maintain its balance. However, when copper levels exceed the body’s regulatory capacity, this could result in copper accumulation in organs such as the liver and brain, which may lead to disorders like Wilson’s disease, a condition characterized by toxic copper buildup. It is, therefore, crucial to monitor copper levels and evaluate the safety of copper ion administration, particularly in combination therapies, to prevent adverse effects from copper overload. In the in vivo experiments, we monitored body weight and general behavior (including activity, grooming, and food intake) and did not observe overt signs of systemic toxicity during TMZ+CuCl_2_ treatment. These observations suggest that the dosing regimen was tolerated at the whole-animal level. However, body weight and behavior alone are relatively coarse indicators of toxicity and do not fully capture potential subclinical copper overload. Future studies will incorporate serum biochemical markers and direct measurements of copper levels in liver and brain tissues to more precisely evaluate copper homeostasis and organ-specific safety. The normal range of serum copper in the human body is about 70–155 μ g/dL, while in mice it is approximately 55–110 μ g/dL.Further studies should address the long-term impact of copper ion treatments, focusing on copper homeostasis and biosafety to better assess the potential risks associated with clinical applications of copper-based therapies in glioma treatment. Interestingly, TMZ+low-dose CuCl_2_ co-treatment enhances the sensitivity of glioma cells to TMZ, leading to a reduction in cell proliferation and migration, and ultimately causing the accumulation of the copper death marker protein DLAT in the mitochondria, triggering copper-induced cell death in glioma cells. This suggests a potential synergistic effect between the two. TMZ blocks copper efflux by downregulating ATP7A, whereas under CuCl_2_ exposure, ATP7A and FDX1 tend to decrease, and the effects are most evident under TMZ+CuCl_2_ co-treatment. ATP7A, a member of the P-type ATPase family, mediates the transmembrane transport of copper ions by consuming energy [23]. It plays a crucial role in maintaining copper homeostasis inside and outside the cell, particularly in extraliver tissues, where it is responsible for transporting cytosolic copper to the basolateral membrane [24]. In the process of intestinal copper absorption and copper transport to the brain tissue, ATP7A is the rate-limiting factor. ATP7A can interact with various adaptor factors, some of which are related to the development of the nervous system [25]. FDX1 encodes a reductase that is known to reduce Cu^2+^ to a more toxic form, Cu^+^. It plays a role in the metabolism of steroid hormones and bile acids, as well as vitamins A and D, and is responsible for transferring electrons to all seven types of Class I mitochondrial cytochrome P450 enzymes [26]. A study by Sun et al. found that the downregulation of FDX leads to ROS-mediated mitochondrial autophagy and activation of the PI3K/AKT signaling pathway, promoting HCC cell proliferation, invasion in vitro, and growth and metastasis in vivo [27]. A study found that FDX1 can regulate the apoptosis and autophagy processes in cells associated with polycystic ovary syndrome [28]. In glioma cells, C-MYC inhibits mitophagy by upregulating FDX1 expression and suppressing the expression of the autophagy marker protein LC3 [8]. However, the individual downregulation of ATP7A or FDX1 is not sufficient to induce cuproptosis, suggesting that other key regulatory factors are involved in the initiation of cuproptosis.

We confirmed through subcutaneous tumorigenesis experiments that TMZ+CuCl_2_ combined treatment significantly inhibited tumor growth. RNA sequencing of the control and TMZ+CuCl_2_ combined treatment groups revealed that TRIM14 was significantly suppressed in the TMZ+CuCl_2_ group. A study found that in endometrial cancer, TRIM14 can activate the classical IκB Kinase (IKK) complex by binding to NF-κB Essential Modulator (NEMO), leading to the phosphorylation of IκBα, releasing NF-κB from its inhibitory state, thereby driving the activation of its signaling pathway and accelerating tumor progression [29]. RNA sequencing results suggest that TMZ+CuCl_2_ co-treatment inhibits several stress- and survival-related pathways, including ubiquitin-mediated protein metabolism, autophagy, Mitogen-Activated Protein Kinase (MAPK), and NF-κB signaling. Western blotting also showed that TMZ+CuCl_2_ treatment reduced the expression of TRIM14 and key non-classical NF-κB pathway proteins p100 and p52. Using TCGA and CGGA databases, we found that TRIM14 is highly expressed in gliomas, and patients with high TRIM14 expression have a poor survival rate. TRIM14 is an E3 ubiquitin ligase, a protein with a molecular mass of approximately 49,773 Da, composed of 442 amino acids, and its encoding gene is located at the human chromosome 9q22.33 region. This protein contains three typical domains: B-box2, coiled-coil domain, and PRY-SPRY domain [30]. Tomar et al. found that TRIM14 can upregulate Dvl2 and form a complex with it, thereby activating the Wnt/β-catenin signaling pathway. This results in increased expression of O-6-Methylguanine-DNA Methyltransferase (MGMT), which can effectively repair alkylation-induced DNA damage, ultimately promoting the development of TMZ resistance in glioblastoma [31]. Liang et al. suggested that the high expression of TRIM14 may play an important role in regulating GBM cell resistance to Histone Deacetylase (HDAC) inhibitors [32].

Our study found that TMZ can suppress the expression of TRIM14 and ATP7A, while it has no significant effect on the expression of ATP7B and SLC31A1. ATP7A is highly expressed in glioma tissues, and patients with high expression of ATP7A have a lower survival rate. Although ATP7B is underexpressed in glioma tissues, and patients with low ATP7B expression have a lower survival rate, its role in copper ion homeostasis is limited. Copper-transporting ATPase 2 (ATP7B) and ATP7A are homologous P-type ATPases [33]. Both enzymes function to transport copper ions from the cytoplasm to cellular compartments with higher copper content, with ATP7B specifically responsible for distributing copper across the liver, brain, and kidney apical membranes [34]. ATP7B is widely expressed in various tissues and plays a key role in regulating copper metabolism in the liver, central nervous system, placenta, and kidneys. Defects in the ATP7B gene lead to Wilson’s disease (WND), characterized by copper accumulation in the liver and brain [35]. Solute Carrier Family 31 Member 1 (SLC31A1) is a major regulator of Cu uptake. This N- and O-glycosylated homotrimeric protein is localized to the plasma membrane, and its large extracellular region is rich in copper-binding amino acids such as histidine and methionine. It is hypothesized that its function is to guide copper ions through the transmembrane channel at the trimer center [36]. Current evidence suggests two potential mechanisms regulate SLC31A1 expression and consequently copper uptake: (1) transcriptional control by Sp1 transcription factor [37] and (2) elevated intracellular copper concentration-triggered endocytosis and subsequent degradation of SLC31A1 protein [38]. Copper ions alone did not suppress TRIM14 expression. TRIM14 typically regulates target gene expression through molecular binding interactions. The PRY-SPRY domain within TRIM14 mediates protein–protein interactions, with studies demonstrating its capacity to upregulate Dvl2 and form stable complexes with this signaling molecule [31]. Ma et al. demonstrated that TRIM14 interacts with NEMO to facilitate IκBα phosphorylation by the canonical IKK complex, leading to NF-κB liberation, pathway activation, and subsequent tumor progression [29]. TRIM14 has been shown to interact with Glutamine: Fructose-6-Phosphate Amidotransferase 1 (GFAT1), the rate-limiting enzyme in the hexosamine biosynthesis pathway (HBP), promoting its ubiquitination-mediated degradation, thereby suppressing HBP flux and exerting tumor-suppressive effects [39]. Our study found that TRIM14 may interact with ATP7A and regulate the expression of ATP7A protein through this interaction (Figure 7). ATP7A is a copper efflux transporter that plays a critical role in the regulation of copper ions inside and outside the cell. Upregulation of ATP7A helps tumor cells maintain copper homeostasis through copper ion efflux, thereby preventing copper-induced cellular damage. ATP7A is a central regulator of copper efflux and an essential determinant of intracellular copper homeostasis. In our study, the transcriptional profiling indicated a marked suppression of TRIM14, a known stabilizer of ATP7A, suggesting that TMZ may indirectly downregulate ATP7A activity and thereby limit copper export. Such a reduction in ATP7A-mediated copper transport would be expected to enhance intracellular copper retention and sensitize glioma cells to copper-dependent cytotoxicity. Although we did not directly manipulate ATP7A in the present study, it is plausible that ATP7A knockdown alone, when combined with copper exposure, could recapitulate the effects observed with TMZ. This possibility raises the intriguing hypothesis that ATP7A represents not only a mechanistic node linking TMZ to copper dysregulation but also a potential therapeutic target. Future work incorporating ATP7A knockdown or pharmacological inhibition will be essential for validating this mechanism and further elucidating the role of copper trafficking in glioblastoma treatment.

In our experiments, TRIM14 overexpression promoted the upregulation of ATP7A, enhancing copper efflux in tumor cells. This may be one of the reasons for its resistance to cuproptosis and TMZ+CuCl_2_ combination therapy (Figure 5). In contrast, TRIM14 knockdown significantly reduced ATP7A expression, leading to copper ion accumulation within the cells and enhancing cuproptosis (Figure 6). This phenomenon suggests that TRIM14 not only helps maintain cellular survival by regulating copper homeostasis but may also promote resistance through the regulation of the cuproptosis pathway.

In this study, we found that TRIM14 regulates cuproptosis by modulating copper homeostasis proteins (such as ATP7A) and lipoylation-related proteins (such as Lipoylated-DLAT and Lipoylated-DLST). When TRIM14 is overexpressed, the upregulation of ATP7A enhances copper efflux, reducing Cu^+^ accumulation and decreasing the aggregation of lipoylated proteins, thereby inhibiting cuproptosis (Figure 5). On the other hand, TRIM14 knockdown increases copper accumulation, enhances lipoylation protein aggregation, and amplifies cuproptosis (Figure 6). This finding supports that cuproptosis is a form of cell death caused by copper homeostasis imbalance, providing a theoretical basis for its potential application in cancer therapy.

TRIM14 not only plays a role in copper homeostasis regulation and resistance mechanisms in glioma but may also influence treatment outcomes by affecting the cuproptosis pathway. Based on the results of this study, targeting TRIM14 may become one of the strategies to enhance cuproptosis and improve drug sensitivity. For example, downregulating TRIM14 expression may enhance the antitumor effects of TMZ+CuCl_2_ co-treatment in gliomas, while avoiding TRIM14′s promotion of resistance through the maintenance of high ATP7A expression.

This study has several limitations. First, we examined only low concentrations of copper ions and did not evaluate the effects or mechanisms of higher copper levels on glioma biology. Second, although p100 and p52 expression were altered, the involvement of the non-canonical NF-κB pathway remains unconfirmed and requires further validation. Third, while we identified a potential interaction between TRIM14 and ATP7A, the precise regulatory mechanism underlying this interaction is still unclear. Additionally, we used a low-dose TMZ-induced “TMZ-insensitive” condition rather than fully established TMZ-resistant glioma cell lines, which may not fully reflect the complexity of clinical TMZ resistance. Future studies using validated resistant models will be necessary to strengthen these findings. Finally, although no overt toxicity was observed in vivo, we did not directly measure serum or tissue copper levels, limiting our assessment of copper homeostasis and potential copper overload. Comprehensive copper quantification and toxicological analyses will be required in future work.

## 5. Conclusions

This study reveals the important role of TRIM14 in glioma and systematically elucidates the potential function of the TRIM14–ATP7A axis in copper homeostasis regulation and cuproptosis. We found that high expression of TRIM14 may enhance glioma cell resistance by promoting the upregulation of ATP7A, reducing copper accumulation, and inhibiting cuproptosis. Conversely, TRIM14 knockdown increases intracellular copper accumulation, promotes cuproptosis, and enhances sensitivity to treatment. Furthermore, the expression patterns of ATP7A and ATP7B significantly affect the prognosis of glioma: high expression of ATP7A is associated with poor clinical prognosis, while low expression of ATP7B is also associated with poor prognosis. Based on these findings, targeting the TRIM14–ATP7A axis may provide a new strategy for glioma treatment, particularly in regulating copper homeostasis and cuproptosis.

## Data Availability

The raw data for each experiment reported can be obtained from the authors upon reasonable request.

## Abbreviations

The following abbreviations are used in this manuscript:

ATP7A	ATPase copper transporting alpha
ATP7B	ATPase Copper Transporting Beta
C-MYC	Cellular myelocytomatosis viral oncogene homolog
CAN	Copy number alterations
CGGA	Chinese Glioma Genome Atlas
co-IP	co-immunoprecipitation
DLAT	Dihydrolipoamide S-acetyltransferase
DMEM	Dulbecco’s Modified Eagle Medium
Dvl2	Disheveled Segment Polarity Protein 2
EMT	Epithelial–mesenchymal transition
FBS	Fetal bovine serum
FDX1	Ferredoxin 1
GBM	Glioblastoma
GFAT1	Glutamine: Fructose-6-Phosphate Amidotransferase 1
HBP	Hexosamine biosynthesis pathway
HDAC	Histone Deacetylase
HGG	High-grade gliomas
IKK	IκB Kinase
LGG	Low-grade gliomas
MAPK	Mitogen-Activated Protein Kinase
MGMT	O-6-Methylguanine-DNA Methyltransferase
NEAA	Non-essential amino acids
NEMO	NF-κB Essential Modulator
NF-κB	Nuclear factor kappa-light-chain-enhancer of activated B cells
PHLPP2	PH Domain and Leucine Rich Repeat Protein Phosphatase
SLC31A1	Solute Carrier Family 31 Member 1
SNV	Single nucleotide variations
TCGA	The Cancer Genome Atlas
TGN	Trans-Golgi network
TMZ	Temozolomide
TRIM	Tripartite motif
TRIM14	Tripartite motif-containing protein 14
TRIM46	Tripartite Motif-Containing 46
WHO	World Health Organization
YTHDF1	YTH domain-containing family protein 1
ZEB2	Zinc Finger E-box Binding Homeobo
